# Integration of ecological indicators to assess a multitemporal impact of cement industries

**DOI:** 10.1007/s11356-024-34079-y

**Published:** 2024-07-18

**Authors:** Claudia Cocozza, Francesco Parisi, Massimo Chiari, Stefano Loppi, Silvana Munzi, Sonia Ravera

**Affiliations:** 1 Department of Agriculture, Food, Environment and Forestry (DAGRI), University of Florence, Via San Bonaventura 13, 50145 Florence, Italy; 2 Department of Biosciences and Territory, University of Molise, C. da Fonte Lappone, 86090 Pesche, IS Italy; 3 NBFC, National Biodiversity Future Center, 90133 Palermo, Italy; 4 INFN Division of Florence and Department of Physics and Astronomy, University of Florence, Via G. Sansone 1, 50019 Sesto Fiorentino, Italy; 5 Department of Life Sciences, University of Siena, Via Pier Andrea Mattioli 4, 53100 Siena, Italy; 6 Centro Interuniversitário de História das Ciências E da Tecnologia, Faculdade de Ciências, University of Lisbon, Campo Grande, 1749-016 Lisbon, Portugal; 7 Center for Ecology, Evolution and Environmental Changes & CHANGE - Global Change and Sustainability Institute, Faculdade de Ciências, University of Lisbon, Campo Grande, 1749-016 Lisbon, Portugal; 8 Department of Biological, Chemical and Pharmaceutical Sciences and Technologies (STEBICEF), University of Palermo, Via Archirafi 38, 90123 Palermo, Italy

**Keywords:** Biomonitoring, Trace elements, Tree rings, Lichens and beetles

## Abstract

The present study evaluated an integrated biomonitoring approach based on three different bioindicators: tree rings, lichens, and beetles in a complex environment (urban-industrial-forest). In Central Italy, four sampling sites were selected to assess the anthropogenic impact of cement plants taking into account (1) long-term exposure (1988–2020) through the analysis of trace elements in tree rings of *Quercus pubescens*; (2) medium-term exposure (2020–2021) through the analysis of trace elements in thalli (outermost portions) of the lichen *Xanthoria parietina*; (3) short-term exposure in spring 2021 through the bioaccumulation and evaluation of sample vitality in transplants of the lichen *Evernia prunastri* and a periodic survey of entomological biodiversity carried out during spring and summer 2021. Trace elements of industrial origin were found in tree rings, with different levels of accumulation between 1988 and 2020 and a maximum in 2012. Native thalli of the lichen *X. parietina* showed an overall low bioaccumulation of trace elements except for Cr, probably reflecting the influence of national lockdown measures. The transplants of *E. prunastri* showed a weak stress response in the industrial and urban sites, but not in the forest, and identified Tl and V as the main elements contributing to atmospheric contamination, with peaks at the industrial sites. Concerning the beetles, a significantly lower number of species was found at the Semonte industrial site.

**Supplementary Information:**

The online version contains supplementary material available at 10.1007/s11356-024-34079-y.

## Introduction

Environmental pollution is a global issue, where anthropogenic impact is the main cause of pollutant availability in the environment (Briffa et al. 2020). Environmental pollution, determined by the accumulation of gases (e.g., NO_*x*_, SO_2_, VOC, and NH_3_) and/or particulate matter (PM), defines significant negative impacts and risks for human and ecosystem health (EEA 2018). Therefore, the monitoring of environmental quality in urban areas is a sensible topic that generates significant interest. Environmental safety is determined by World directives, such as the World Health Organization’s (WHO) guidelines, and by National Emission Ceilings Directive that regulate environmental quality monitoring through regional environmental agencies (WHO 2017; De Marco et al. 2019). Traditional air quality monitoring is widely available with each country housing environmental agencies tasked with this responsibility (https://aqicn.org/sources/). Nevertheless, several factors prevent the complete data collection necessary for the establishment of suitable policies. In some cases, monitoring stations are set upped to only measure specific pollutants and/or in defined periods not continuous. Moreover, the resource-intensive maintenance of instruments requires significant financial investment that may generate the reduction of the quality of data acquisition.

Biomonitoring relies on the sensitivity of biological organisms (bioindicators) that interact with the environment where they live to gather valuable information (Costa and Teixeira 2014). Plants and animals are continuously exposed to environmental conditions that regulate their growth, physiology, productivity, and distribution (Malmstrom 2010). Although data interpretation in biomonitoring can be a complex undertaking, biomonitoring offers numerous advantages as a valuable tool for ecological and human health monitoring, primarily due to the widespread availability of bioindicators.

Several organisms are currently used as bioindicators, among which were tree rings (Ballikaya et al. 2022), lichens (Abas 2021), and beetles (Parisi et al. 2018). According to current dendrochemistry applications, trees serve as effective monitor of pollutants, offering data for past decades (Binda et al. 2021; Ballikaya et al. 2022). Trees are subjected to continuous exposure to trace elements, which can be taken up through their roots, leaves, and bark. These elements are subsequently deposited in tree rings, allowing for the decoding of environmental signals corresponding to each year of wood formation through the dendrochemistry approach (Perone et al. 2018; Cocozza et al. 2021). Lichens, given their close dependence on the atmosphere for water supply and mineral nutrients, are sensitive to the presence of substances that alter the normal atmospheric composition. Trace elements can be absorbed directly through the surface of thalli and accumulated (e.g., Vannini et al. 2019; Anderson et al. 2022) thus indicating differences in the elements’ availability during their exposure to the environment. Insects, abundant and widely distributed in all habitats, can act as bioaccumulators and indicators of air pollution (Gutiérrez et al. 2020). Their response can be observed through alterations in life cycle duration, mortality rate, and overall abundance. Trees, lichens, and insects that grow in urban areas, namely in parks and gardens, ensure an effective distribution in the landscape, which can compensate for the lack of both historical pollution time series and artificial air monitoring networks (Baroni et al. 2023).

While biomonitoring studies usually focus on one specific taxon, a combined approach proved to be effective in forest (Burrascano et al. 2021, and reference therein) and urban environment (Pinho et al. 2016). The integrated response of several biological groups can reveal temporal and spatial patterns of environmental variables since different ecological indicators, with varied characteristics, respond differently to human disturbance and can be present in the study sites in different times.

The study was aimed to detect the trace elements in a complex urban environment by using three different bioindicators. The sampling of different biomonitors was designed to consider different durations of exposure in the environment, namely, long-term (ca. 30 years) exposure in wood (trees cores), medium-term (ca. one year) exposure in native foliose lichens, and short-term exposure in insects (beetles sampled from June to October 2021) and lichen transplants (exposed from April to July 2021). Lichens and tree cores are used as bioaccumulators, where bioaccumulation refers to the process whereby a substance present in the environment accumulates at the surface of an organism and/or penetrates it (ISPRA Guidelines, Giordani et al. 2020).

Four different sites were selected by identifying as potential sources of environmental pollution two cement plants, listed by EEA (Holland et al. 2011) among the industries with the greatest environmental and health impact in Europe. Thus, an urban, a forest, and two different industrial sites were considered.

## Materials and methods

### Study area

The study area is in the Gubbio Plain, located in Central Italy (43°21′ N, 12°34′ E, 495 m a.s.l.). This extensive intermontane basin includes a valley region and the foothills of the Monti di Gubbio, situated to the north of the town. The primary sources of air pollutants (i.e., NO_*x*_, SO_*x*_, As, Cd, Cr, Pb, and Ni) are two operational cement plants. Other relevant sources of air pollutants (CO, VOCs, PM_2.5_, and PM_10_) are heating systems, followed by vehicular traffic (mostly CO and NO_*x*_) and agriculture (the principal source of NH_3_).

**Fig. 1:**
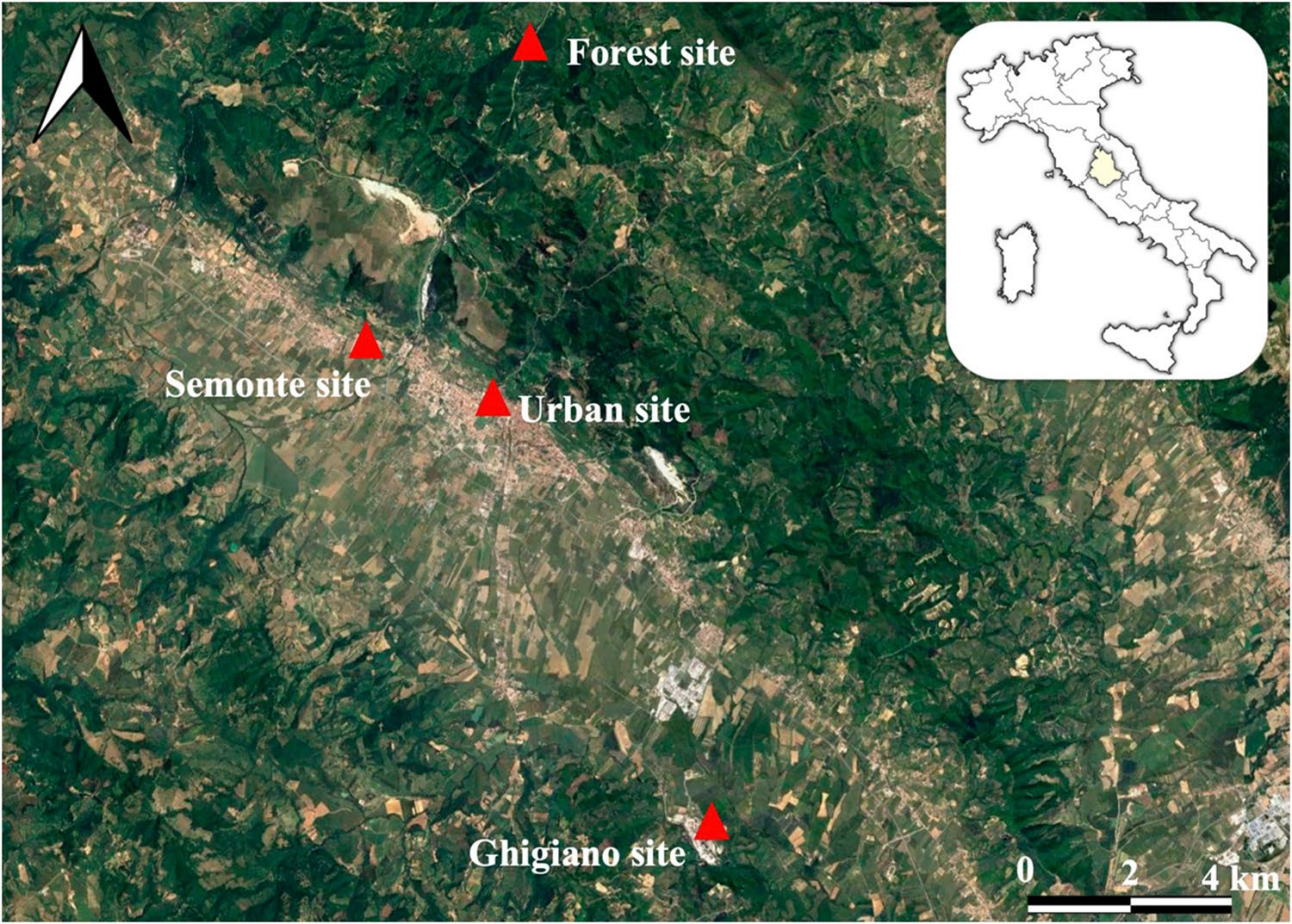
Localization of sampling sites in the study area: the cement plant site (Ghigiano), the urban cement plant site (Semonte), urban (Parco Ranghiasci), and forest (S. Bartolomeo) sites

The first cement plant, the Ghigiano site, is a manufacturing facility established in 1966, located 8 km away from the urban center of Gubbio. The amount of cement produced in the plant in the Ghigiano site is around 4 million tons per year (https://www.colacem.com/media/userfiles/files/Colacem_RS_2021(2).pdf). The second cement plant, the Semonte site, houses a cement factory built in 1926 and is located less than 2 km from downtown Gubbio, along a high-traffic regional road, with a cement production of 1.3 million tons per year. Besides those two sites, the study area includes an urban site, Parco Ranghiasci, a park located downtown within a limited traffic zone, which was chosen as the reference for the urban environment. Finally, a forest site, namely, a control site, known as S. Bartolomeo, was selected in a rural environment situated 7 km away from the town center and Semonte site and 15 km away from the Ghigiano site. The S. Bartolomeo Forest site was specifically chosen to characterize environmental conditions that are less influenced by urban disturbances (Fig. 1).

Data on the deposition of some airborne pollutants in the study area are available at https://apps.arpa.umbria.it/webgis/emissioni/index.asp.

### Sampling

Tree cores, lichens, and insects were sampled at the four sampling sites: Ghigiano and Semonte (industrial), Parco Ranghiasci (urban), and S. Bartolomeo (forest).

Three trees of *Quercus pubescens* Willd. characterized by a mean diameter at breast height (DBH) of 28 ± 3 cm and average age of 30 ± 2 years were selected in four sampling sites. Two cores per tree were collected at DBH by using an incremental borer (Haglof Company Group, Sweden) in April 2021 and then cut using a microtome (Gärtner and Nievergelt 2010). The tree ring width was measured using the LINTAB instrument (Rinntech, Heidelberg, Germany) and a Leica MS5 stereoscope (Leica Microsystems, Germany). The raw tree-ring width chronologies were obtained by the software TSAP Win (Rinn 1996) and then statistically cross-dated to identify the year of tree-ring formation (Speer 2012). The Gleichläufigkeit statistical index and the relative significance value were calculated (Schweingruber 1988; Speer 2012). The signature of the temporal evolution of environmental conditions was obtained over a long period through the trace element measurements in tree rings from 1988 to 2020 (the common period of tree ring chronologies within sampling sites).

To consider medium- and short-term exposure, the lichen species were chosen among those most widely used in biomonitoring studies (e.g., Cocozza et al. 2016; Vannini et al. 2017; Contardo et al. 2021). Regarding medium-term exposure, fragments of the foliose lichen *Xanthoria parietina* (L.) Th. Fr., were collected in April 2021 from the tree trunks used for dendrochemical analysis, following ISPRA Guidelines (Giordani et al. 2020). Specifically, the outermost portions (3 mm), corresponding to about 1 year of metabolic activity (Tretiach and Carpanelli 1992), were selected to obtain three analytical samples for each site.

To consider a short-term exposure, thalli of the fruticose lichen *Evernia prunastri* (L.) Ach. were collected from deciduous oak trees in a forested area of the Umbria Region (Stroncone), far from any local pollution sources. Prior to transplantation, extraneous residues such as bark and insects were removed from the thalli. Sample vitality was then randomly checked by analyzing the photosynthetic efficiency. Lichen thalli were transplanted at the four sampling sites (three samples per plot), suspended to the branches of the trees used for the dendrochemical analysis, following ISPRA Guidelines (Giordani et al. 2020). An exposure of 12 weeks is regarded as optimal for *E. prunastri* (Loppi et al. 2019).

In the four sampling sites, the capture of saproxylic and non-saproxylic adult beetles was performed. Forest beetle community was considered because saproxylic species are closely related to tree wood (Speight 1989) and include threatened taxa (see, Carpaneto et al. 2015), whereas non-saproxylic beetles refer to all remaining species occupying other ecological niches. Many of the non-saproxylic species occupy unknown trophic niches (Audisio et al. 2015). The beetle sampling was carried out using four window flight traps (WFTs) per site for flying beetles suspended to the branches of the trees used for the dendrochemical analysis (in total 16 traps). At each site, the WFTs were located approximately 10 m from each other. Traps were checked four times, approximately every 30 days, from June to October 2021 and then removed. Systematics and nomenclature followed Bouchard et al. (2011).

### Trace element analysis

The tree core samples were measured with particle-induced X-ray emission (PIXE) technique at the INFN LABEC ion beam laboratory in Florence (Chiari et al. 2021), which is located a 3 MV Tandetron accelerator. A 3.00 MeV proton beam was used, extracted into atmosphere through a 200-nm-thick Si_3_N_4_ membrane. Beam size was defined by a 1.0-mm-diameter collimator, placed in vacuum before the extraction window. The tree core samples were positioned roughly at 1 cm from the beam exit window (the proton beam energy on the sample surface was 2.95 MeV considering the energy loss in the Si_3_N_4_ window and the path in external atmosphere) and moved by micrometric stepper motors to allow aiming at a specific tree ring. Beam current intensity, indirectly measured by means of a rotating chopper (Chiari et al. 2002), varied between 3 and 4 nA (to keep dead time at few percent at most) and the measurements lasted 300 s each. Two X-ray detectors were used for PIXE measurements, a 10 mm^2^ (collimated to 3.5 mm^2^) Silicon Drift Detector (SDD), 4.5 cm from sample with He flow in front in order to reduce the absorption of lower energy X-rays in air, for light elements (Na–Ca) analysis, and a 150 mm^2^ SDD, 2 cm from sample, with 450 μm Mylar absorber, for heavy (> Ca) and trace elements. The collected PIXE spectra were then analyzed with the GUPIXWin software package (Campbell et al. 2010) using the trace element solution option, applying an instrumental parameter obtained by the analysis of X-ray spectra of external reference standards (NIST 1412 and NIST 610). The analysis of tree rings was performed in latewood, more uniform than earlywood in oak (Perone et al. 2018), and in one ring every four to obtain a consistent signal and check it within the intra-ring variability (Ballikaya et al. 2023) by collecting data from 1988 to 2020. The analysis defined trace element concentrations in earlywood of tree rings formed in 1988, 1992, 1996, 2000, 2004, 2008, 2012, 2016, and 2020.

The trace element concentrations to be compared within sampling sites were normalized, using the following equation:

$${value}_{\text{normalized}}=\left({value}_{x}-{value}_{\text{lowest}}\right)/\left({value}_{\text{highest}}-{value}_{\text{lowest}}\right)$$

where *value*_*x*_ refers to the level of a specific year, and *value*_lowest_ and *value*_highest_ refer to the lowest and the highest element concentrations, respectively, measured in tree rings of each site.

To determine trace elements in lichens, samples of *X. parietina* and *E. prunastri* were frozen, powdered and homogenized by grinding in a mill with Teflon balls. Concerning *E. prunastri*, transplants (three samples, each composed by 300 mg of powdered lichen material, for the control and each site) were analyzed in the University of Siena. Samples were mineralized with a mixture of 3 mL of 70% HNO_3_, 0.2 mL of 60% HF, and 0.5 mL of 30% H_2_O_2_. Digestion of samples was carried out in a microwave digestion system (Milestone Ethos 900) for a total time of 30 min. Concentrations of trace elements (Ba, Ce, Co, Cr, Cu, Dy, Fe, Ga, I, Mn, Nb, Ni, Pb, Pr, Rb, Sr, Tb, Tl, U, V, Zn, Zr), expressed on a dry weight basis, were determined by inductively coupled plasma mass spectrometry (ICP-MS, Perkin-Elmer Sciex Elan 6100) using the “Total Quant” method with both standard (STD) and KED (kinetic energy dispersion) operating modes. This method was chosen in order to have the widest possible overview of the trace elements present in the study area at the time of the research. One procedural blank and one sample of the certified material GBW-07604 “Poplar leaves” were also analyzed.

The values of bioaccumulation in lichens, namely, the ratio between species-specific element concentration values in (i) native samples of *X. parietina* and the corresponding background element concentration values (B-Ratio) and (ii) exposed *E. prunastri* samples and the corresponding element concentration values measured in unexposed samples (EU-Ratio) was calculated.

The interpretation of the ratios followed ISPRA Guidelines (Giordani et al. 2020). The attribution of a sampling site *i* to a class of the bioaccumulation scale (i.e., absence of bioaccumulation, low bioaccumulation, moderate bioaccumulation, high bioaccumulation, severe bioaccumulation) has to be performed on the basis of the mean value of the *B* ratio or EU ratio diminished by its uncertainty (e.g., for transplants: EU^(i)^ − Δ(EU^(i)^).

### Chlorophyll a fluorescence measurements

Lichen vitality was assessed in thalli of the lichen *E. prunastri* before and after a 12-week exposure in the study area by measuring the chlorophyll *a* fluorescence emission with a plant efficiency analyzer fluorimeter (Hand PEA, Hansatech, Norfolk, UK). The maximum quantum yield of primary photochemistry calculated as *Fv*/*Fm* = (*Fm* – *F*0)/*Fm*, where *F*0 and *Fm* are minimum and maximum chlorophyll *a* fluorescence and *Fv* = (*Fm* − *F*0) is the variable fluorescence, and the photosynthetic performance *PI*_ABS_, a global indicator of the photosynthetic performance, were measured (Strasser et al. 2004). The fluorescence parameters were determined in hydrated thalli (five measurements for each of three samples for a total of 15 measurements for the control and each site), after 10 min of dark adaptation, applying a saturating flash of light of 2400 µmol s^−1^ m^−2^ for 1 s.

### Statistical analysis

Descriptive statistics (means, standard errors) were calculated for all the measured trace elements in tree rings and lichens. One-way ANOVA with post hoc Tukey HSD test was applied to test the effect of site in environmental signal. Time series of the trace elements were analyzed through the Kruskal–Wallis test to test significant differences between the index levels of elements over time in relation to concentration in years. To assess the relationships among trends in different elements and investigate spatial pollution patterns, principal component analyses (PCA) were applied to trace elements. PCA is an ordination technique to project onto several dimensions, generally two, defined by the axes of maximal variance (Hammer and Harper 2006). The principal components with eigenvalues greater than 1.0 were retained. Statistical analyses were performed using OriginPro 8 program (OriginLab Corporation, Northampton, UK).

## Results

### Tree rings

Mean tree-ring chronologies ranged from 1988, 1986, 1978, and 1988 to 2020 in Ghigiano, Semonte, forest, and urban sites, respectively. The mean tree-ring width was 3.42 mm (± 0.15 mm) (Table 1). Cross-dating between site-mean chronologies showed a Gleichläufigkeit value of 67 (*p* < 0.05) (Table 1).

**Table 1:** Characteristics of the tree ring width chronology (*P* < 0.05)

Site	Period	Raw mean ring width (mm) of chronology	Standard deviation (mm)	Glk
Ghigiano site	1988–2020	2.68	0.17	66
Semonte site	1986–2020	3.11	0.16	67
Forest site	1978–2020	3.85	0.14	69
Urban site	1988–2020	4.05	0.15	65

**Fig. 2:**
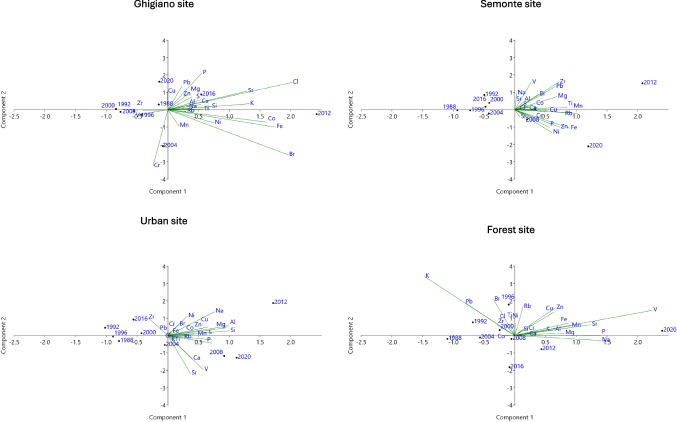
Biplot diagrams from the principal component analysis of elements detected in tree rings of *Q. pubescens* on spatial (Ghigiano, Semonte, forest, and urban sites) and temporal scale (1988, 1992, 1996, 2000, 2004, 2008, 2012, 2016, 2020). The biplot in the first and second component planes is shown with the elements (dots) and parameters (vectors) measured. Each dot indicates the average for the three samples

Trace elements detected in the tree rings by PIXE analysis were Al, Br, Ca, Cl, Co, Cr, Cu, Fe, K, Mg, Mn, Na, Ni, P, Pb, Rb, S, Si, Sr, Ti, V, Zn, and Zr (Table 2). Concentrations of Al, Mg, Mn, and P resulted to be differently distributed within measured tree rings and sampling sites (Table 2). The ordination diagram (I and II components), resulting from PCA applied to biomonitors (wood), was grouped in four sampling sites (Ghigiano, Semonte, urban, and forest sites) for the measured tree rings (Fig. 2). The PCA defined an element ordination in tree rings of *Q. pubescens* in Ghigiano, Semonte, and urban sites in 2012 that was not observed in the forest site (Fig. 2). The ordination PCA defined 23 PCs: the first component accounted for over 31% and the second for over 16%; two grouping of elements were obtained: the first group of elements was constituted by Al, Ca, Cu, Mg, Na, P, S, Si, Sr, and Zn and the second group by Br, Cl, Cr, Fe, K, Mn, Ni, Pb, Rb, Ti, V, and Zr (Figure S1). Moreover, the ordination PCA allowed to define representative elements in tree rings of *Q. pubescens* for each sampling site: Ghigiano site (Br, Ca, Cl, Cr, Cu, Mg, Ni, P, Pb, Rb, V, Zn and Zr), Semonte site (Al, Br, Cl, Cu, Fe, K, Mg, Mn, Na, P, Si, Ti and Zn), urban site (Al, Ca, Cr, Cu, Fe, Mg, Mn, Ni, P, S, Sr, Ti, and Zn), and forest site (Br, Cl, Cu, Fe, K, Mg, Mn, Na, P, Pb, Rb, Ti, V, Zn, and Zr) (Fig. S1). Therefore, elements were identified as differently distributed between sampling sites, except for Cu, Mg, P, and Zn which were found in all sites.

**Table 2:** Concentrations (mg/g dry weight) of trace elements in the tree rings (mean values ± standard errors) in Ghigiano, Semonte, urban, and forest sites

Tree-ring	Ghigiano site	Semonte site	Urban site			Forest site	Kruskal–Wallis
	Mean st.er	Mean st.er	Mean st.er			Mean st.er	
	**Al**						**0.024**
1988	87.70 ± 12.9	17.4 ± 0.8	49.0 ± 2.5			-	
1992	83.12 ± 25.6	147 ± 6	-			180 ± 89	
1996	150 ± 76	148.0 ± 17.0	18.6 ± 0.9			148 ± 45	
2000	35.19 ± 22.1	84.5 ± 11.6	22.1 ± 1.1			18.9 ± 2.2	
2004	105 ± 97	203.2 ± 12.3	185 ± 9			211 ± 116	
2008	132 ± 100	461 ± 147	663 ± 33			55 ± 8	
2012	252 ± 91	458 ± 133	1082 ± 54			93 ± 5	
2016	285 ± 168	476.5 ± 20.9	54.4 ± 2.7			105 ± 42	
2020	147 ± 128	159 ± 34	426.7 ± 21.3			427 ± 121	
	**Br**						**0.230**
1988	0.60 ± 0.11	-	-			0.38 ± 0.03	
1992	0.2064 ± 0.003	0.153 ± 0.027	0.264 ± 0.013			0.67 ± 0.05	
1996	0.631 ± 0.09	0.291 ± 0.028	- ± -			0.93 ± 0.17	
2000	0.31 ± 0.07	0.25 ± 0.07	0.395 ± 0.020			0.24 ± 0.08	
2004	1.220 ± 0.4	0.32 ± 0.06	0.270 ± 0.013			0.46 ± 0.05	
2008	0.74 ± 0.25	-	0.113 ± 0.006			0.31 ± 0.07	
2012	1.648 ± 0.3	1.11 ± 0.27	0.62	±	0.03	0.156 ± 0.023	
2016	0.64 ± 0.24	0.19 ± 0.04	-			-	
2020	-	-	-			0.257 ± 0.020	
	**Ca**						**0.224**
1988	2295 ± 53	2895 ± 354	5286 ± 264			1310 ± 140	
1992	491.1 ± 12.4	1655 ± 892	1710 ± 86			895 ± 313	
1996	1257 ± 819	1873 ± 257	3178 ± 159			1615 ± 736	
2000	794 ± 271	1594 ± 511	2083 ± 104			1050 ± 524	
2004	629 ± 275	933 ± 246	5805 ± 290			966 ± 433	
2008	966 ± 282	1660 ± 234	6114 ± 306			2013 ± 735	
2012	3060 ± 742	3691 ± 884	3368 ± 168			1350.5 ± 25.8	
2016	1205 ± 401	1134 ± 285	4413 ± 221			1036 ± 231	
2020	1650 ± 535	2096 ± 70	10,322 ± 516			2759 ± 1342	
		**Cl**					**0.824**
1988	141 ± 44	14.81 ± 10.5	-			-	
1992	55 ± 32	27 ± 6	-			50.9 ± 18.5	
1996	62.6 ± 20.7	19 ± 8	10.0 ± 0.5			91.8 ± 11.5	
2000	37.3 ± 22.9	14 ± 5	43.3 ± 2.2			61 ± 9	
2004	71.2 ± 23.3	3.7 ± 2.6	-			37.9 ± 20.7	
2008	45.26 ± 10.1	57 ± 7	29.2 ± 1.5			67.0 ± 25.2	
2012	278 ± 70	39.7 ± 9	18.8 ± 0.9			35.3 ± 1.0	
2016	337 ± 61	41 ± 4	36.2 ± 1.8			20.4 ± 8	
2020	143 ± 82	19.41 ± 1.1	27.8 ± 1.4			3.4 ± 2.4	
		**Co**					**0.763**
1988	2.8 ± 0.7	2.4 ± 0.6	1.50 ± 0.08			-	
1992	3.4 ± 1.0	1.0 ± 0.6	2.52 ± 0.13			1.163 ± 0.021	
1996	4.3 ± 1.6	1.34 ± 0.23	1.81 ± 0.09			0.43 ± 0.10	
2000	3.2 ± 1.6	1.160 ± 0.1	2.77 ± 0.14			1.60 ± 0.25	
2004	4.9 ± 2.9	2.06 ± 0.04	5.24 ± 0.26			1.292 ± 0.013	
2008	2.5 ± 0.7	4.08 ± 0.07	4.60 ± 0.23			-	
2012	8.9 ± 1.1	4.3 ± 0.9	4.82 ± 0.24			0.77 ± 0.09	
2016	3.9 ± 1.7	1.07 ± 0.07	2.79 ± 0.14			0.83 ± 0.11	
2020	3.81 ± 1.4	1.42 ± 0.23	3.21 ± 0.16			-	
		**Cr**					**0.6**
1988	16.2 ± 14.0	2.42 ± 0.12	0.77 ± 0.04			0.81 ± 0.12	
1992	43 ± 50	2.0 ± 0.4	10.0 ± 0.5			9 ± 5	
1996	26.2 ± 28.6	10.7 ± 0.4	2.42 ± 0.12			6.4 ± 2.8	
2000	23.4 ± 25.9	16.1 ± 0.6	2.40 ± 0.12			2.0 ± 1.2	
2004	114 ± 139	4.7 ± 1.5	8.596 ± 0.4			7 ± 3	
2008	14.6 ± 17.8	19.0 ± 0.8	10.1 ± 0.5			1.2 ± 0.9	
2012	21.9 ± 13.1	26.9 ± 2.1	14.8 ± 0.7			8.3 ± 1.4	
2016	9 ± 6	2.80 ± 0.11	18.10 ± 0.9			0.53 ± 0.27	
2020	10.0 ± 1.4	57 ± 5	3.53 ± 0.18			22 ± 5	
		**Cu**					**0.17**
1988	2.1 ± 0.5	1.9 ± 0.5	2.03 ± 0.10			0.659 ± 0.015	
1992	2.4 ± 2.1	1.057 ± 0.010	2.00 ± 0.10			4.0 ± 1.0	
1996	1.7 ± 0.8	0.61 ± 0.14	2.68 ± 0.13			2.6 ± 0.7	
2000	4.1 ± 1.3	1.8 ± 0.5	3.16 ± 0.16			1.06 ± 0.05	
2004	3.0 ± 1.7	1.3 ± 0.5	2.48 ± 0.12			1.5 ± 0.5	
2008	4.3 ± 1.4	2.2 ± 0.4	4.60 ± 0.23			1.77 ± 0.12	
2012	3.2 ± 1.2	3.7 ± 0.5	6.1 ± 0.3			1.86 ± 0.24	
2016	3.3 ± 1.2	1.7 ± 0.5	2.39 ± 0.12			1.20 ± 0.08	
2020	5.0 ± 2.6	3.0 ± 0.5	3.49 ± 0.17			3.5 ± 0.3	
		**Fe**					**0.059**
1988	274 ± 75	217 ± 38	159 ± 8			69.6 ± 14.3	
1992	521 ± 93	121.0 ± 24.3	196 ± 10			361 ± 205	
1996	534 ± 263	113 ± 39	155 ± 8			572 ± 344	
2000	416 ± 194	256 ± 146	215.4 ± 10.8			240 ± 67	
2004	1039 ± 253	441 ± 188	471.9 ± 23.6			315.0 ± 14.9	
2008	653 ± 316	1658 ± 204	887 ± 44			159 ± 66	
2012	2090 ± 1651	1483 ± 228	451.9 ± 22.6			664.7 ± 17.2	
2016	595 ± 415	117 ± 30	273.8 ± 13.7			43.8 ± 2.8	
2020	624 ± 199	2201 ± 146	170 ± 9			1049 ± 230	
		**K**					**0.635**
1988	2457 ± 132	1458 ± 208	1612 ± 81			2801 ± 305	
1992	1892 ± 813	1281 ± 117	1464 ± 73			2731 ± 180	
1996	1811 ± 761	1219 ± 250	1783 ± 89			3179 ± 132	
2000	1507 ± 590	1139 ± 286	1664 ± 83			2525 ± 99	
2004	1362 ± 497	1035 ± 212	1856 ± 93			2432 ± 147	
2008	1269 ± 117	978 ± 319	1664 ± 83			1899.5 ± 17.3	
2012	3013 ± 481	1415 ± 279	1774 ± 89			1232 ± 143	
2016	1712 ± 232	708 ± 196	1392 ± 70			770 ± 30	
2020	1662 ± 363	1447 ± 159	1857 ± 93			1127 ± 142	
		**Mg**					**0.013**
1988	96 ± 30	153 ± 8	224.1 ± 11.2			168 ± 7	
1992	155 ± 95	310 ± 102	-			269.2 ± 10.4	
1996	165 ± 140	107 ± 7	6.8 ± 0.3			348 ± 56	
2000	43 ± 34	94.9 ± 20.5	67 ± 3			64.7 ± 27.2	
2004	187 ± 183	138 ± 31	335.7 ± 16.8			322 ± 114	
2008	205 ± 98	378 ± 78	490.5 ± 24.5			155 ± 75	
2012	292.1 ± 23.7	1171 ± 132	1232 ± 62			314.9 ± 20.5	
2016	345 ± 117	176 ± 94	87.9 ± 4			313 ± 95	
2020	604 ± 239	276 ± 30	766 ± 38			683 ± 52	
		**Mn**					**0.009**
1988	2.6 ± 1.8	1.30 ± 0.26	2.76 ± 0.14			5.0 ± 0.5	
1992	5.7 ± 2.6	0.106 ± 0.016	3.08 ± 0.15			5.6 ± 0.6	
1996	5 ± 3	1.9 ± 1.0	3.76 ± 0.19			7.0 ± 0.5	
2000	4.4 ± 2.4	4.4 ± 1.7	3.80 ± 0.19			4.6 ± 1.4	
2004	14 ± 4	1.03 ± 0.16	8.1 ± 0.4			4.4 ± 2.3	
2008	4.6 ± 2.6	6.7 ± 1.7	8.4 ± 0.4			5.2 ± 1.1	
2012	7.3 ± 2.7	25 ± 4	14.2 ± 0.7			6.2 ± 1.2	
2016	4.5 ± 1.2	3.2 ± 1.1	5.01 ± 0.25			3.4 ± 0.4	
2020	8.5 ± 2.2	21.0 ± 0.7	11.9 ± 0.6			15.9 ± 0.5	
		**Na**					**0.088**
1988	40.1 ± 28.4	64 ± 37	57.7 ± 2.9			-	
1992	324.9 ± 19.3	613 ± 124	-			136.3 ± 24.7	
1996	106 ± 32	85.5 ± 13.4	-			104 ± 9	
2000	205 ± 37	58 ± 7	413.1 ± 20.7			130 ± 5	
2004	375.2 ± 2.9	298 ± 7	-			-	
2008	151 ± 42	27.0 ± 0.6	-			28 ± 5	
2012	344 ± 74	359 ± 42	854 ± 43			141.1 ± 20.6	
2016	177.4 ± 12.0	338.6 ± 29.8	318.6 ± 15.9			289 ± 31	
2020	482.4 ± 14.6	-	563.9 ± 28.2			600 ± 43	
		**Ni**					**0.438**
1988	1.14 ± 0.11	0.89 ± 0.27	1.02 ± 0.05			1.38 ± 0.18	
1992	2.4 ± 0.7	0.93 ± 0.06	1.00 ± 0.05			1.28 ± 0.20	
1996	2.2 ± 0.6	0.32 ± 0.06	1.21 ± 0.06			2.7 ± 0.7	
2000	1.7 ± 0.8	0.55 ± 0.18	1.24 ± 0.06			1.1 ± 0.4	
2004	3.0 ± 1.7	1.14 ± 0.27	1.32 ± 0.07			2.43 ± 0.03	
2008	2.3 ± 1.1	2.9 ± 0.6	2.68 ± 0.13			0.53 ± 0.12	
2012	4.6 ± 1.7	2.40 ± 0.21	5.35 ± 0.27			1.16 ± 0.14	
2016	1.9 ± 0.7	0.58 ± 0.20	4.96 ± 0.25			0.79 ± 0.11	
2020	1.7 ± 0.3	8.2 ± 1.0	1.21 ± 0.06			1.65 ± 0.25	
		**P**					**0.009**
1988	18 ± 7	-	163 ± 8			-	
1992	15.5 ± 0.9	36.4 ± 1.8	118 ± 6			36.9 ± 14.1	
1996	56.8 ± 12.3	13 ± 9	171 ± 9			45 ± 5	
2000	15.4 ± 10.6	25.7 ± 0.7	233.1 ± 11.7			49.4 ± 2.3	
2004	65 ± 7	92 ± 43	472.0 ± 23.6			87.1 ± 16.5	
2008	94 ± 31	102.0 ± 13.6	388.8 ± 19.4			109.4 ± 21.5	
2012	217 ± 85	249 ± 33	546.3 ± 27.3			124 ± 4	
2016	179 ± 31	98.1 ± 15.9	315.9 ± 15.8			73.5 ± 12.0	
2020	644 ± 181	674 ± 53	766 ± 38			525.6 ± 19.7	
		**Pb**					**0.352**
1988	1.00 ± 0.12	-	1.16 ± 0.06			1.2 ± 0.3	
1992	2.4 ± 0.5	0.64 ± 0.16	1.24 ± 0.06			1.65 ± 0.18	
1996	1.70 ± 0.21	0.26 ± 0.12	-			1.6 ± 0.4	
2000	1.3 ± 0.4	1.03 ± 0.17	0.89 ± 0.04			1.07 ± 0.15	
2004	- ± -	0.28 ± 0.05	-			1.27 ± 0.21	
2008	0.7 ± 0.3	0.55 ± 0.17	-			0.55 ± 0.09	
2012	1.9 ± 0.3	3.29 ± 0.28	0.89 ± 0.04			-	
2016	1.2 ± 0.3	0.22 ± 0.06	-			0.24 ± 0.03	
2020	1.57 ± 0.24	-	0.95 ± 0.05			0.31 ± 0.07	
		**Rb**					**0.755**
1988	1.94 ± 0.24	1.32 ± 0.2	0.347 ± 0.017			1.8 ± 0.4	
1992	2.5 ± 1.6	0.75 ± 0.07	0.76 ± 0.04			1.46 ± 0.15	
1996	1.69 ± 0.19	0.66 ± 0.21	0.88 ± 0.04			2.46 ± 0.27	
2000	1.5 ± 0.8	0.935 ± 0.022	0.61 ± 0.03			1.92 ± 0.25	
2004	1.8 ± 0.5	1.99 ± 0.24	-			0.282 ± 0.028	
2008	1.7 ± 0.9	1.00 ± 0.21	1.71 ± 0.09			1.94 ± 0.25	
2012	2.3 ± 1.1	3.6 ± 0.5	1.13 ± 0.06			1.55 ± 0.27	
2016	1.9 ± 0.3	1.0 ± 0.4	0.73 ± 0.04			0.59 ± 0.17	
2020	1.6 ± 0.4	3.10 ± 0.07	1.11 ± 0.06			1.60 ± 0.21	
		**S**					**0.466**
1988	106.3 ± 15.6	66.6 ± 11.6	164.8 ± 8			44 ± 8	
1992	79 ± 31	49.9 ± 13.4	138.5 ± 7			84.2 ± 14.1	
1996	135.2 ± 21.6	61.1 ± 17.1	122.7 ± 6			112.7 ± 12.0	
2000	104.9 ± 21.9	74 ± 6	201.9 ± 10.1			77 ± 3	
2004	127 ± 31	105 ± 7	283.3 ± 14.2			121.7 ± 14.7	
2008	97 ± 36	69.9 ± 16.1	274.5 ± 13.7			82.1 ± 22.9	
2012	170.8 ± 14.8	129.4 ± 27.3	323.9 ± 16.2			105 ± 8	
2016	127 ± 45	82.0 ± 18.5	228.5 ± 11.4			80 ± 8	
2020	203 ± 39	75.0 ± 10.6	352.2 ± 17.6			145 ± 39	
		**Si**					**0.062**
1988	54 ± 9	55 ± 5	135 ± 7			32 ± 9	
1992	73.1 ± 29.6	133.3 ± 29.3	209.2 ± 10.5			131 ± 53	
1996	207 ± 36	69 ± 9	69 ± 3			190.6 ± 21.0	
2000	83 ± 8	69 ± 37	123 ± 6			88.9 ± 11.8	
2004	91 ± 34	280 ± 37	363.4 ± 18.2			100 ± 9	
2008	143 ± 72	1028 ± 100	1029 ± 51			68 ± 8	
2012	503 ± 451	179.8 ± 23.3	1322 ± 66			70 ± 7	
2016	84.5 ± 26.2	671.4 ± 17.4	145 ± 7			81.0 ± 11.2	
2020	274 ± 70	308.3 ± 22.4	703 ± 35			161.6 ± 12.3	
		**Sr**					**0.49**
1988	16.4 ± 2.3	6.2 ± 0.3	13.2 ± 0.7			5.3 ± 1.1	
1992	4.0 ± 2.5	4.6 ± 1.1	5.87 ± 0.29			7.3 ± 1.7	
1996	7.0 ± 2.1	4.9 ± 1.2	10.5 ± 0.5			11.1 ± 1.0	
2000	6.5 ± 1.1	2.2 ± 0.8	4.96 ± 0.25			6.9 ± 0.5	
2004	5 ± 4	4.2 ± 1.2	17.9 ± 0.9			5.4 ± 0.8	
2008	10 ± 4	5.4 ± 0.4	22.2 ± 1.1			10.1 ± 0.9	
2012	17 ± 3	9.7 ± 0.5	5.43 ± 0.27			9.78 ± 0.27	
2016	12 ± 3	3.3 ± 1.4	7.9 ± 0.4			7.2 ± 1.8	
2020	10.1 ± 2.1	2.015 ± 0.019	21.1 ± 1.1			17.6 ± 0.5	
		**Ti**					**0.369**
1988	2.075 ± 0.028	4.3 ± 0.6	7.1 ± 0.4			5.8 ± 0.5	
1992	4.7 ± 0.8	1.2 ± 0.7	-			15.4 ± 1.2	
1996	3.4 ± 0.7	1.88 ± 0.24	-			5.4 ± 1.2	
2000	2.1 ± 0.4	3.4 ± 1.0	3.36 ± 0.17			3.0 ± 0.9	
2004	3.4 ± 2.4	4.0 ± 0.6	10.1 ± 0.5			3.4 ± 1.5	
2008	1.9 ± 1.3	13 ± 4	14.6 ± 0.7			3.3 ± 0.8	
2012	10 ± 4	30 ± 3	5.49 ± 0.27			5.7 ± 0.7	
2016	4.0 ± 2.1	4.1 ± 0.9	9.8 ± 0.5			2.1 ± 0.6	
2020	4.8 ± 1.6	15.9 ± 0.9	0.71 ± 0.04			6.7 ± 2.3	
		**V**					**0.199**
1988	0.810 ± 0.014	-	-			-	
1992	1.0 ± 0.6	1.58 ± 0.06	-			-	
1996	1.9 ± 1.2	0.26 ± 0.06	-			0.98 ± 0.08	
2000	0.67 ± 0.09	1.8 ± 0.3	1.25 ± 0.06			1.307 ± 0.011	
2004	1.19 ± 0.08	-	-			0.324 ± 0.006	
2008	1.53 ± 0.18	0.86 ± 0.05	1.79 ± 0.09			0.80 ± 0.26	
2012	0.923 ± 0.024	1.77 ± 0.17	-			0.93 ± 0.24	
2016	0.37 ± 0.11	1.21 ± 0.10	-			-	
2020	1.59 ± 0.10	-	1.86 ± 0.09			2.32 ± 0.22	
		**Zn**					**0.227**
1988	2.8 ± 0.4	1.47 ± 0.24	1.60 ± 0.08			-	
1992	2.1 ± 0.7	0.42 ± 0.11	3.07 ± 0.15			1.58 ± 0.27	
1996	1.3 ± 0.5	0.62 ± 0.05	3.87 ± 0.19			5.02 ± 0.22	
2000	1.9 ± 1.4	1.16 ± 0.12	3.36 ± 0.17			1.9638 ± 0.0020	
2004	1.9 ± 1.6	0.99 ± 0.22	4.86 ± 0.24			2.13 ± 0.25	
2008	2.6 ± 1.0	2.70 ± 0.14	6.3 ± 0.3			2.55 ± 0.28	
2012	3.0 ± 1.0	4.40 ± 0.07	7.3 ± 0.4			1.65 ± 0.18	
2016	3.2 ± 2.1	1.51 ± 0.21	4.26 ± 0.21			1.4 ± 0.4	
2020	3.2 ± 1.4	7.6 ± 0.6	4.18 ± 0.21			3.5 ± 0.6	
		**Zr**					**0.185**
1988	1.13 ± 0.10	1.03 ± 0.04	-			-	
1992	1.0 ± 0.7	0.915 ± 0.027	0.73 ± 0.04			0.84 ± 0.07	
1996	-	-	-			-	
2000	-	-	-			1.22 ± 0.17	
2004	0.7 ± 0.5	-	-			-	
2008	-	-	-			-	
2012	-	3.30 ± 1.7	-			-	
2016	-	-	2.80 ± 0.14			-	
2020	0.7 ± 0.8	-	-			-	

Kruskal–Wallis was applied to test significant differences between the concentrations of elements in tree rings (*p* level values are given)

**Fig. 3:**
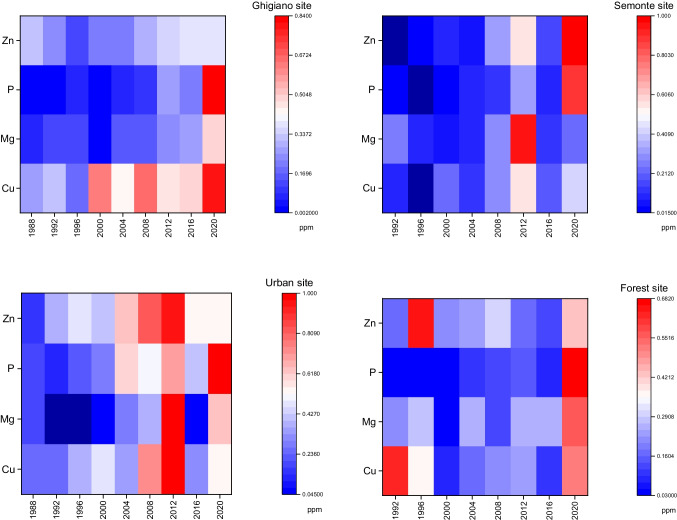
Heat map resuming the representative elements (Cu, Mg, P, Zn, the common elements detected in four sites) in tree rings of *Q. pubescens* for each sampling site, Ghigiano, Semonte, urban, and forest sites, obtained by PCA elements grouping (Fig. S1). Values are mean normalized data of element concentrations in tree-rings (standard error is < 0.1). The highest values are represented by dark red, whereas the lowest values are represented by dark blue

By observing the common elements (Cu, Mg, P, Zn) found in four sites, the highest normalized concentrations were found in 2012 in Ghigiano, Semonte, and urban sites (Fig. 3). A common high levels of elements were in 2020 in four sites, while Cu and Zn resulted higher in 1992 and 1996, respectively, in forest sites than others (Fig. 3).

### Native lichens

The analysis of *X. parietina* samples revealed the presence of Cr, Cu, Fe, Mn, Ni, and Zn in the lichen thalli, with a low level of bioaccumulation of Cr in the industrial sites and of Zn in the urban site (Table 3). The Ghigiano site exhibited the highest concentration of Cr, which was 1.4 times higher than the concentration observed at the forest site, along with elevated levels of Fe. At the Semonte site, the highest concentrations of Mn and Ni were recorded, while the forest site showed the highest levels of Cu and Zn.

**Table 3:**
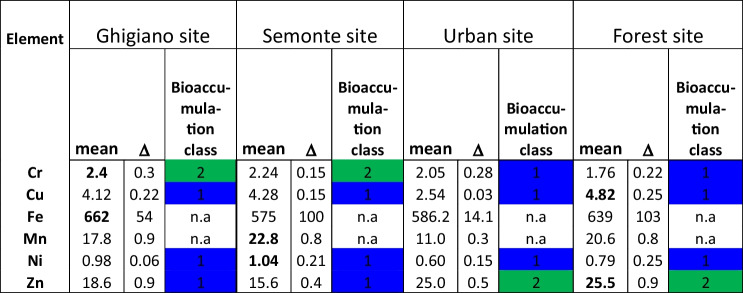
Trace elements concentrations (μg g-1 dry weight) (mean and uncertainty values) in *X. parietina,* bioaccumulation class in Ghigiano, Semonte, urban, and forest sites

Colours associated with bioaccumulation class follow ISPRA 2020 (green: low accumulation, blue: absence of bioaccumulation)

### Lichens transplants

After 12 weeks of exposure to the environmental conditions of the sampling sites, all elements presented a certain degree of bioaccumulation at least in one sampling site, except for Dy and Rb (Table 4). Mostly, the elements showed a low level of bioaccumulation while a moderate bioaccumulation was found for Ce and Fe in Ghigiano, Zr in the forest site, Pr in Ghigiano and the urban sites, and U and V in Semonte and the urban site (Table 4). High bioaccumulation of V was observed only at the Ghigiano site, while severe bioaccumulation of Tl, the main air pollutant in the area among the considered elements, was found in both industrial sampling sites of Ghigiano and Semonte (Table 4).

**Table 4:**
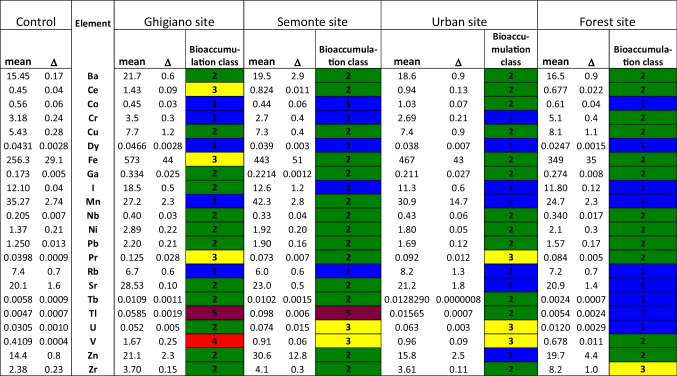
Trace element concentrations (μg g^−1^ dry weight) (mean and uncertainty values) in the lichen transplants, bioaccumulation class in control Ghigiano, Semonte, urban, and forest sites

Colours associated with bioaccumulation class follow ISPRA 2020 (green: low bioaccumulation, blue: absence of bioaccumulation, yellow: moderate bioaccumulation, red: high bioaccumulation, Tyrian purple: severe bioaccumulation)

### Photosynthetic efficiency of lichen transplants

**Fig. 4:**
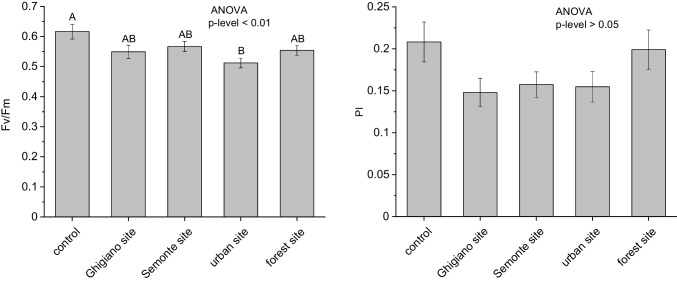
Physiological parameters of *E. prunastri*, *F*_*v*_/*F*_*M*_, and PI (*N* = 15; mean ± standard deviation), in control samples and sampling sites after 12 weeks of exposure: Ghigiano, Semonte, urban, and forest sites. One-way ANOVA defines the significance of the effects of the site. Different letters indicate statistical differences between sites at *P* < 0.05

After 12 weeks of exposure to the environmental conditions of the sampling sites, the physiological parameter of *E. prunastri* were lower than the control in all sites. However, *Fv*/*Fm* showed a statistically significant decrease with respect to the control value only in the urban site (− 17%, *p* level = 0.012) while the *PI*_ABS_ was significantly lower in the industrial and urban sites (up to − 29% with respect to the control) but not in the forest site (Fig. 4).

### Insects

The abundance of specimens, species, and threatened categories for each site was identified (Table 2). All the taxa collected during the field activities are alphabetically listed in Table 5 together with the proportion of the collected species, grouped according to the prevalent trophic categories. We collected 478 specimens belonging to 57 species referring to 23 families of Coleoptera (Table 5). Species strictly considered as saproxylic (sensu Carpaneto et al. 2015) are also reported in Table 5, together with their risk category at the Italian level (see Carpaneto et al. 2015). The beetle assemblages were grouped according to the prevalent trophic categories, defined by Carpaneto et al. (2015). The most abundant species were *Cetonia aurata* and *Protaetia morio* (Cetoniidae), respectively, with 94 and 80 specimens. Regarding the IUCN risk categories, the sampled saproxylic beetles were classified as follows: near threatened (NT; 3 species) and least concern (LC; 18 species). Xylophagous and saproxylophagous accounted for 5.7% of the total sampled species, followed by Sap-feeder (4%), predators (2.28%), and only two specimens of mycophagous were collected.

**Table 5:** Number of beetle specimens, species, and species included in Red List categories—near threatened (NT) in sampling sites: Ghigiano, Semonte, urban and forest sites

Beetles	Ghigiano site	Semonte site	Urban site	Forest site	ANOVA
					*p* level
Specimen	113b	245a	65b	55b	0.004
Specie	23a	7b	27a	29a	0.001
Specie—NT category	1	0	1	1	

One-way ANOVA defines the significance of the effects of the site. Different lowercase letters indicate statistical differences between sites at *P* < 0.05

## Discussion

### Environmental signal by trees

The study revealed distinct patterns of trace element accumulation in tree rings across time and space. The detection of trace elements in tree rings provided insight into the availability of several elements (Al, Br, Ca, Cl, Co, Cr, Cu, Fe, K, Mg, Mn, Na, Ni, P, Pb, Rb, S, Si, Sr, Ti, V, Zn, and Zr) in the environment, information that was not previously available. The variability in tree ring composition over time allowed the assessment of temporal patterns of pollutant accumulation in different sampling sites. The bioaccumulation of elements in tree rings presents some challenges in pinpointing the exact source, as the production of certain pollutants cannot be attributed exclusively to specific anthropogenic activities. However, compelling evidence of trace elements in tree rings was found in relation to the industrial activity of the study area. Chlorine and Br were detected in both the Ghigiano and Semonte sites. Chlorine is known as a potentially harmful element typically derived by the combustion of waste-derived fuel (e.g., Gerassimidou et al. 2021), while Br is used as halogen additives to reduce mercury emissions (UNEP 2011). In the Ghigiano site, relevant concentrations of Cr, Cu, Pb, Rb, and V were found, indicating the area as a potential source of heavy metals and metalloids, a major threat to environmental and human health (Fan et al. 2021). In the Semonte site, the presence of Cu, Fe, Mg, Mn, Si, Tl, and Zn confirmed the existence of a contamination chain like heavy metals, metalloids, and “”non-metals, usually released from industries into the atmosphere, soil, water, and contaminating food (Pourret et al. 2021). Signals were detected in tree rings in 2012 in industrial and urban sites by highlighting the pollution signals characteristic of antropizated ecosystems, deriving from anthropogenic contamination and traffic emission (Bibi et al. 2023). On the other hand, the forest site showed elements characteristics with different time frame. The common element accumulation in tree rings of 2020 on four sites might be attributed to various production processes, including combustion in manufacturing industries, energy and transformation industries, and residential combustion (Capon and de Saulles 2023), suggesting a process of elements’ diffusion. However, it is worth noting that extremely toxic and hazardous metals like As and Hg (Index 2018) were not found in the tree rings in any of the sampling sites in Gubbio.

### Environmental signal by lichens

The dual approach used for lichens provided information pertaining to two distinct time spans: 1 year, approximately corresponding to 2020—first months of 2021, in the case of native samples collected in situ and a shorter subsequent period (spring 2021) in the case of the transplants.

The analysis of thalli of *X. parietina* revealed enrichment of Cr in both industrial sites, which has been associated with the impact of cement production in previous studies (Paoli et al. 2017). An enrichment in Zn was found in the urban and forest sites and the latter also showed the highest concentration of Cu. The occurrence of these elements can be attributed to agricultural practices, primarily linked to the olive groves surrounding the forest site. Indeed, insecticides and fungicides often contain Cu and Zn, like the Bordeaux mixture (CuSO_4_) and the Mancozeb® (Zn) (National Center for Biotechnology Information 2017).

When examining the medium-term contamination of the study area, it is important to consider the significant reduction in air pollution that occurred in 2020 resulting of the widespread lockdown measures implemented to curb the spread of the coronavirus infection (Donzelli et al. 2020; Ravina et al. 2021). This reduction in air pollution could be a contributing factor to the relatively low levels of trace element bioaccumulation (e.g., Tl which is instead highly accumulated in lichen transplants) observed in the lichen material corresponding to this period. However, it was not observed in tree rings perhaps due to the selectivity of element absorption regulated by root, for example.

The analysis of the transplants identified Tl among the measured elements, as the primary contributing factors to atmospheric contamination in the study area. These findings agree with similar studies carried out around cement factories (Demiray et al. 2012; Gallo et al. 2014; Paoli et al. 2014, 2017). In fact, Tl can be present as an impurity in raw materials, and its compounds are volatile at high temperatures (ATSDR 1992) making cement factories significant anthropogenic sources of this element (IPCS 1996). Vanadium appears to be predominantly associated with combustion activities that involve heavy petroleum products, e.g., petcoke used as main fuel by the two cement factories (as reported in their 2021 Environmental Performance Indicators). Although it is possible that residential combustion contributed to some extent to atmospheric contamination in the urban site, previous studies have shown that high concentrations of V are primarily linked to the use of petcoke as fuel in plant factories (e.g., Gallo et al. 2014), and V and Tl have been identified as reliable markers of cement factory activity. In addition, among the trace elements bioaccumulated by transplants, a few lanthanoids (Ce, Pr, Tb) have been also detected. The accumulation of lanthanoids in mosses and lichens is commonly attributed to the deposition of soil dust (Wu et al. 2020), along with Fe. The presence of Zr, a traffic-related element (Lyubomirova et al. 2011), and U in the study area is likely attributed to the same origin, namely, the resuspension of soil particles from the many dirty roads in the surroundings. The presence of U is explained by the natural radioactivity of the study area (data from Regione Umbria, available at: https://www.regione.umbria.it/documents/18/20164312/radioattivita+naturale/086b8f8b-82ec-476a-8894-66eb5f013d87?t=1586461453107). The effect of a high-traffic road near the industrial site of Semonte is suggested by the higher concentrations of Mn and Zn, in agreement with similar studies carried out in urban areas (Paoli et al. 2013; Wu et al. 2020).

Transplanted lichen thalli were still alive at the end of the exposure period, confirming the ongoing functionality of the active component within the bioaccumulation process.

A decrease in fluorescence parameters is a non-specific physiological response to stress factors. In our study, this alteration can be likely attributed to the increased availability of pollutants since the samples transplanted to the forest site did not show significant difference from the control samples. A comparable reduction of the chlorophyll *a* fluorescence emission in transplants of *E. prunastri* has been recorded in the center of Milan (Contardo et al. 2021).

### Environmental signal by beetles

The beetle community was not characterized to demonstrate environmental pollution. Beetles were characterized by wood-related populations and the presence of threatened species in urban environments (e.g., Forister et al. 2019; Kotze et al. 2022; Kitahara and Fujii 1997). Usually, these species are very sensitive to disturbances and can be valid indicators of environmental health. The beetle community sampled at the four sites showed differences both in terms of specimens and species collected. In particular, the Semonte and Ghigiano sites included most of the specimens collected. The Semonte site was integrated into an agroecosystem with oak trees arranged along a road and agricultural fields. The abundance of specimens attributable to a few species is typical of over-anthropized ecosystems where human activities have an impact on biodiversity levels (Chowdhury et al. 2023). As it is known in the scientific literature, natural environments that are little subject to human activities present constant levels of biodiversity. In fact, in the forest site, the relationship between the number of beetle species and trophic categories (i.e., predators and prey) seems to be in balance (see Table 5). A similar relationship, between predators and prey, was observed in the Ghigiano site (abandoned oak forest) and in the urban site. In the latter, the trees in the site (mainly holm oaks) probably favored a good presence of species despite the urban park management activities (Sabatelli et al. 2023

## Conclusions

Combined biomonitoring generally might offer several advantages in environmental assessment and monitoring compared to single-taxon biomonitoring. Different species have varying sensitivities and ecological roles, making them indicators of diverse aspects of environmental quality. Furthermore, the use of different organisms helps overcome the limitations of relying solely on a single group, which might not be present in the entire study area. This work highlights how combining the response of taxonomic groups with different life cycles can provide information about environmental conditions across various time spans or at specific moments. In a complex environment like the study area, including several ecosystems and pollution sources, this approach provides a more comprehensive and nuanced understanding of environmental conditions and changes. The sampling design highlighted the different effects of anthropized ecosystems to element diffusion in the environment. In detail, the sampling in forest sites resulted necessary to isolate signal of industrial and urban activities. Integrating data from various biomonitoring approaches, despite discrepancies, offers a holistic understanding of environmental changes, identifies pollution sources, and informs conservation and management efforts. Additionally, such studies should focus on providing data for mapping and visualizing biomonitoring, aiding in raising awareness, guiding land management decisions, and supporting conservation strategies. However, to be effective, this combined approach requires support from multiple case studies, and consistent and numerous sampling sites are essential.

### Supplementary Information

Below is the link to the electronic supplementary material.

Supplementary file1 (DOCX 557 KB)

Supplementary file2 (XLSX 15 KB)

## Data Availability

All data generated or analyzed during this study are included in this published article.
