# Supplementary material for: Integration of ecological indicators to assess a multitemporal impact of cement industries
Source: Environ Sci Pollut Res Int. 2024 Jul 18;31(35):48233–49. doi: 10.1007/s11356-024-34079-y (PMC11297839; doi:10.1007/s11356-024-34079-y)

**Supplementary materials**

**Table S1.** List of species of saproxylic and non saproxylic beetles and number of specimens collected in sampling sites in Gubbio.

*in Tables S1.xlsx*

**Figure S1.** Biplot diagrams from the Principal Component Analysis obtained by the grouping of elements of Fig. 2. The grouping of elements in the dial at the left of Fig. 2 (Al, Ca, Cu, Mg, Na, P, S, Si, Sr, Zn) was exploited in the biplot diagram A (Al, Cu, Mg, Na, Si) and B (Ca, P, S, Sr, Zn), the grouping of elements in the dial at the right of Fig. 2 in C (Cr, Fe, Mn, Ni, Ti) and D (Br, Cl, K, Pb, Rb, V, Zr), data obtained by analysis of tree rings of *Q. pubescens* grown in Ghigiano, Semonte, forest and urban sites.

B


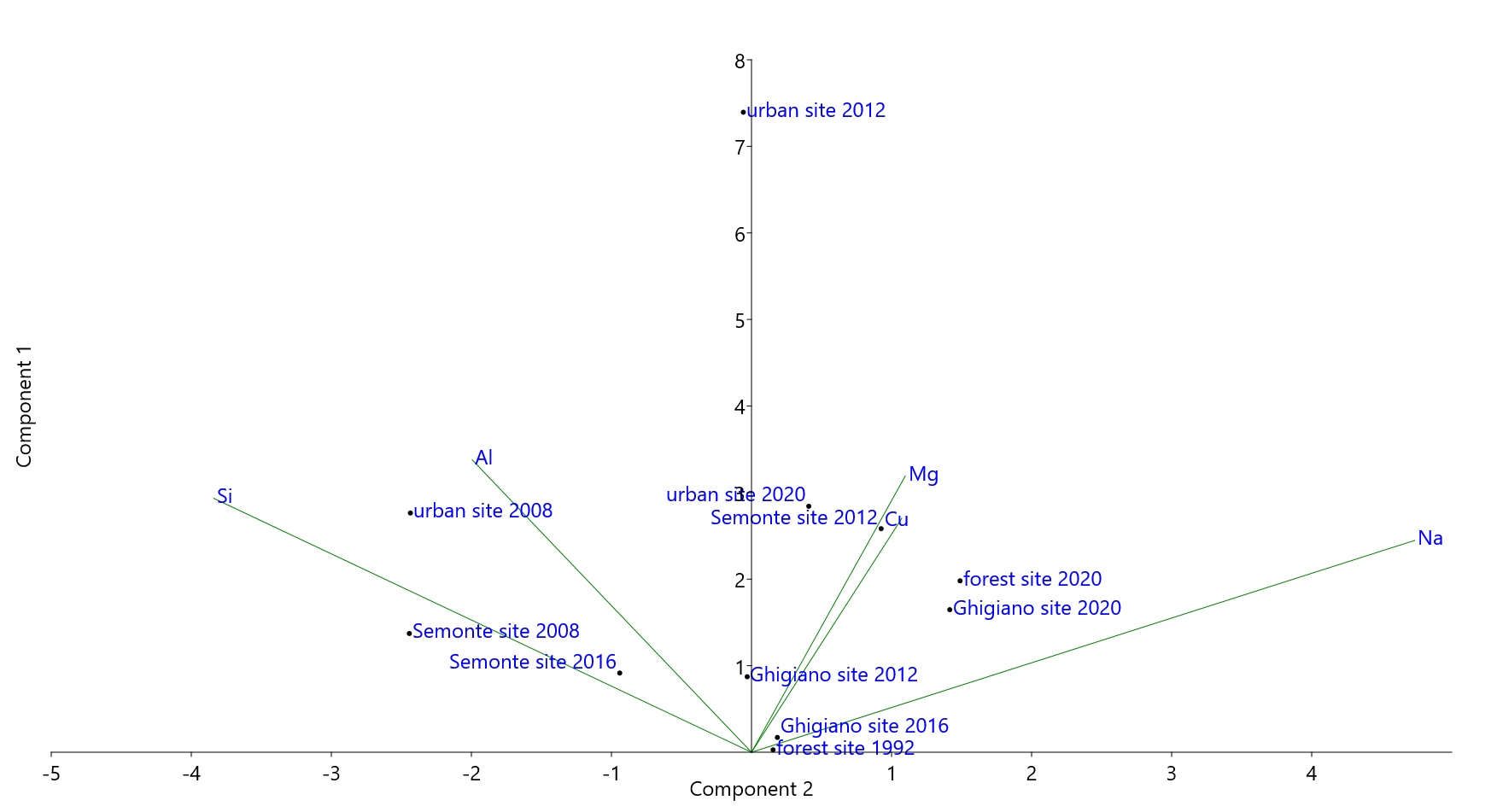

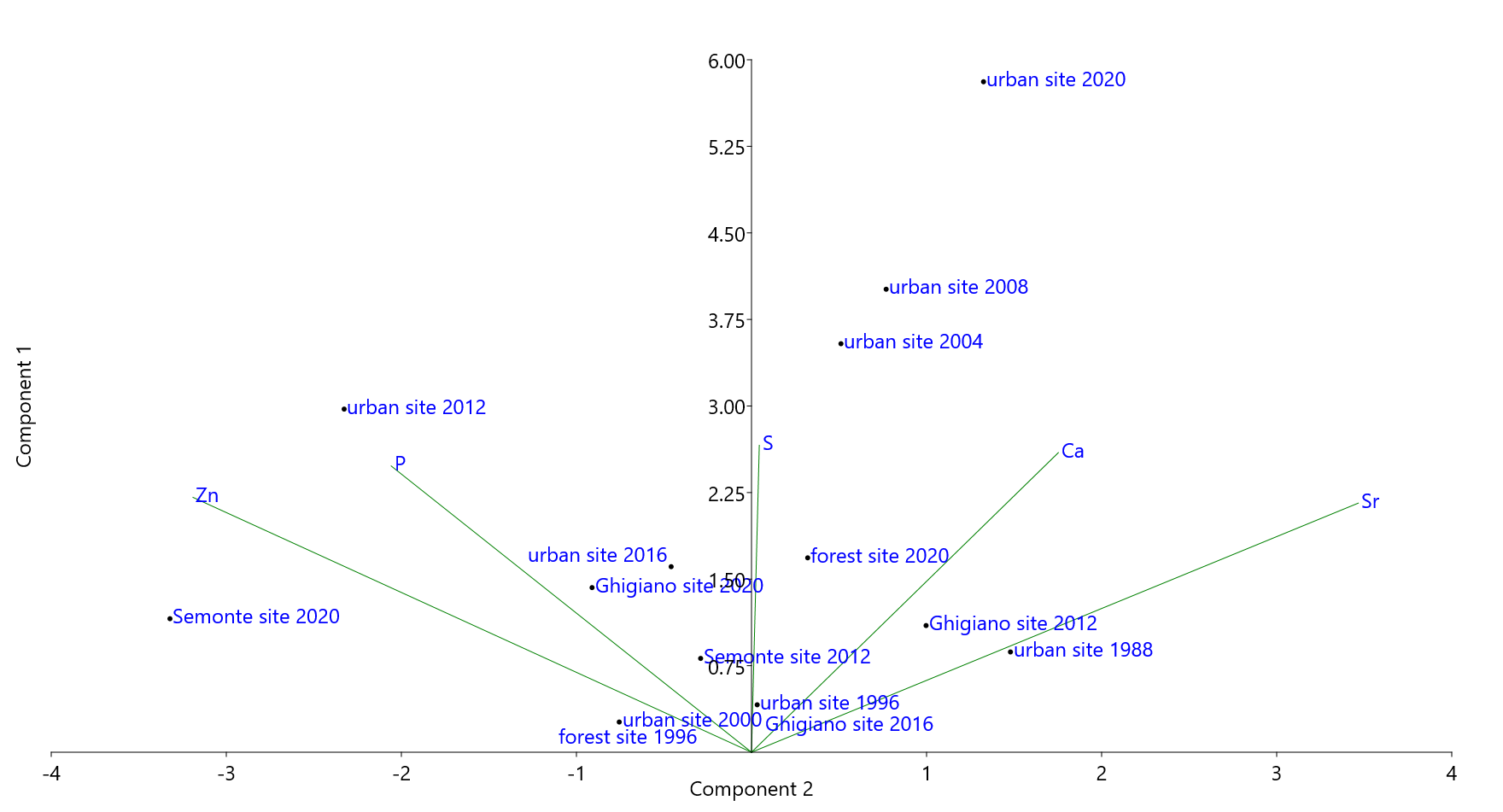

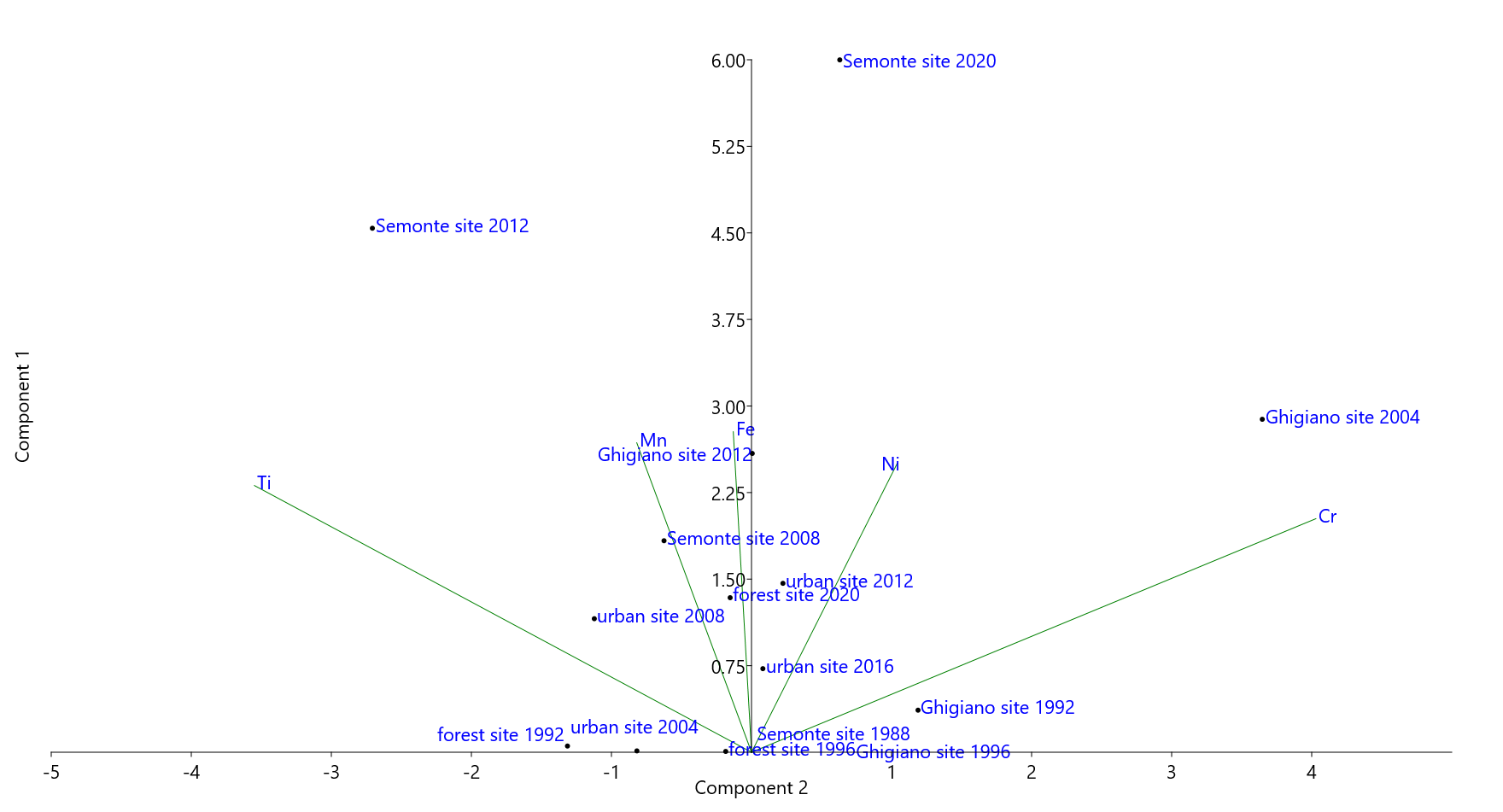

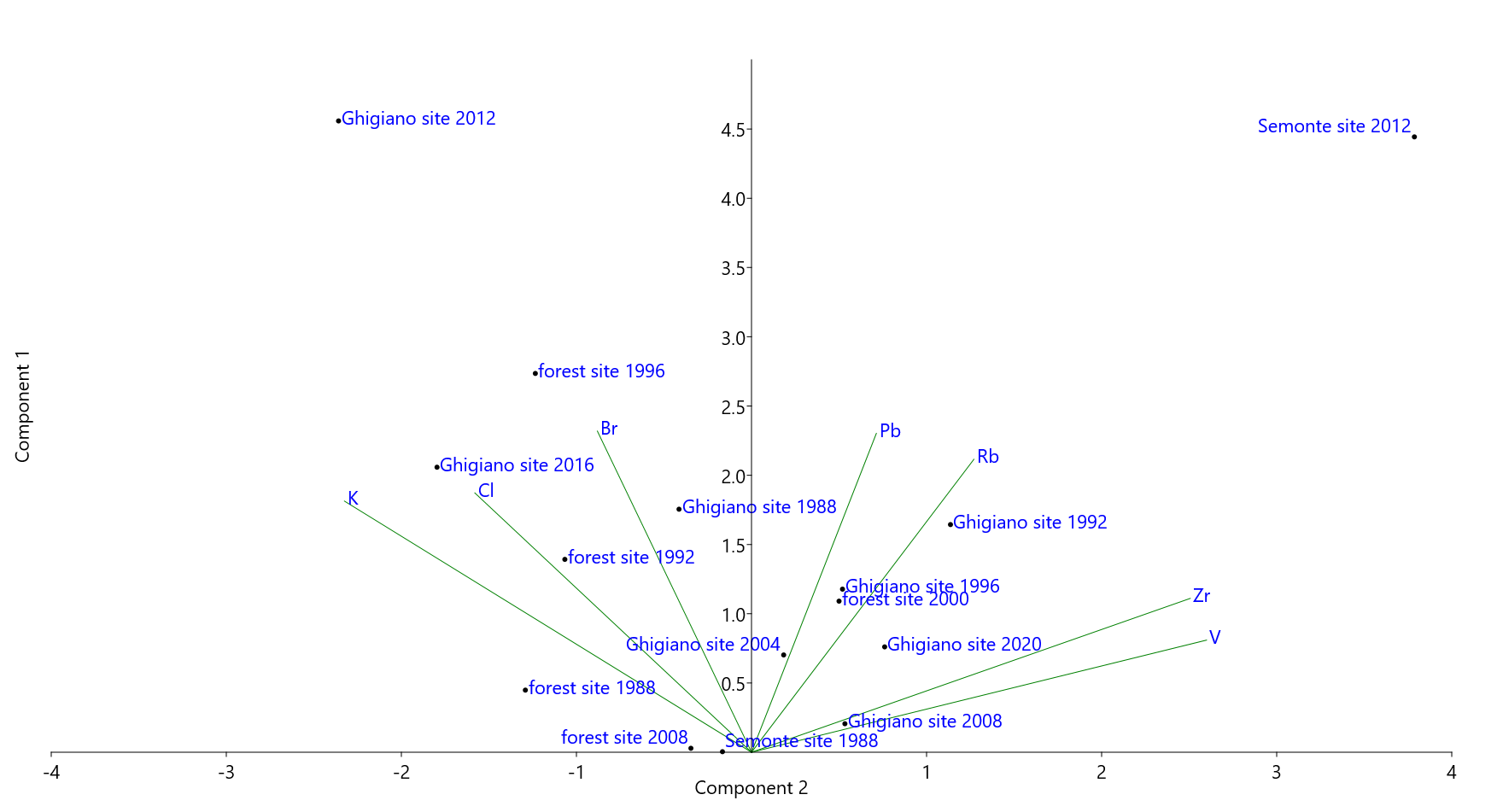


A

C

D

**Fig. S2.** Heat map resuming the representative elements in tree rings of *Q. pubescens* for each sampling site, Ghigiano, Semonte, urban and forest sites, obtained by PCA elements grouping (**Fig. S1**). Values are mean normalized data of element concentrations in tree-rings (standard error is < 0.1). The highest values are represented by dark red, whereas the lowest values are represented by dark blue.


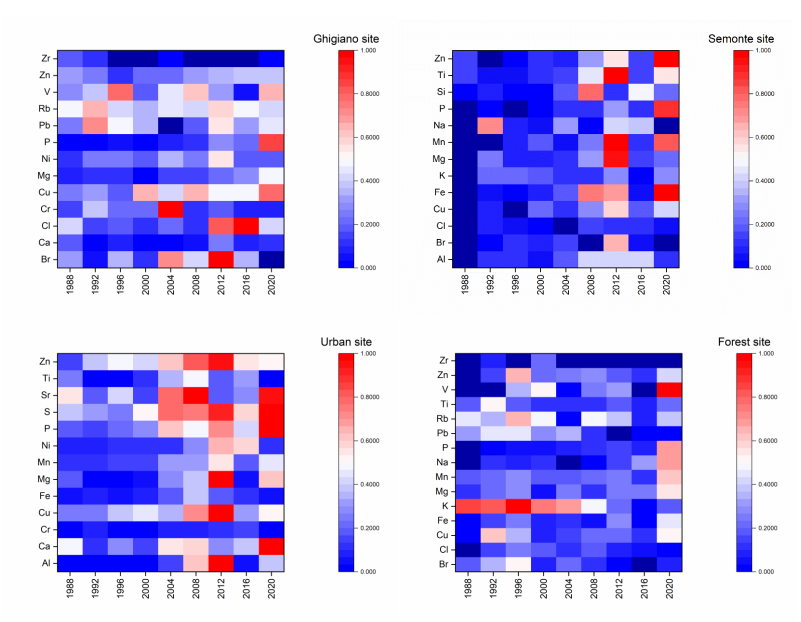

Supplement: Supplementary file 1 — Supplementary file1 (DOCX 557 KB) [file 11356_2024_34079_MOESM1_ESM.docx]
